# Structure and Function of Bovine Whey Derived Oligosaccharides Showing Synbiotic Epithelial Barrier Protective Properties

**DOI:** 10.3390/nu12072007

**Published:** 2020-07-06

**Authors:** Peter I. Duncan, Olli Aitio, Annamari Heiskanen, Ritva Niemelä, Juhani Saarinen, Jari Helin, Nadine Porta, Muriel Fiaux, Denis Moënnoz, Mireille Golliard, Christine Cherbut, Rafael Berrocal, Sean Austin, Norbert Sprenger

**Affiliations:** 1 Nestlé Institute of Health Sciences, Société des Produits Nestlé S.A., 1000 Lausanne, Switzerland; peter.duncan@rdls.nestle.com (P.I.D.); nadine.porta@rdls.nestle.com (N.P.); muriel.fiaux@rfsm.ch (M.F.); mireille.golliard@rdls.nestle.com (M.G.); christine.cherbut@inrae.fr (C.C.); 2 Glykos Finland Ltd., 00790 Helsinki, Finland; olli.aitio@glykos.fi (O.A.); annamari.heiskanen@glykos.fi (A.H.); ritva.niemela@glykos.fi (R.N.); juhani.saarinen@glykos.fi (J.S.); jari.helin@glykos.fi (J.H.); 3 Nestlé Product Technology Center, Société des Produits Nestlé S.A., 3510 Konolfingen, Switzerland; rbf1952@gmail.com; 4 Nestlé Institute of Food Safety and Analytical Sciences, Société des Produits Nestlé S.A., 1000 Lausanne, Switzerland; sean.austin@rdls.nestle.com

**Keywords:** epithelial barrier function, lactobacillus probiotic, host–microbiota interaction

## Abstract

Commensal gut microbiota and probiotics have numerous effects on the host’s metabolic and protective systems, which occur primarily through the intestinal epithelial cell interface. Prebiotics, like galacto-oligosaccharides (GOS) are widely used to modulate their function and abundance. However, important structure–function relations may exist, requiring a detailed structural characterization. Here, we detailed the structural characterization of bovine whey derived oligosaccharide preparations enriched with GOS or not, dubbed GOS-enriched milk oligosaccharides (GMOS) or MOS, respectively. We explore GMOS’s and MOS’s potential to improve intestinal epithelial barrier function, assessed in a model based on barrier disruptive effects of the *Clostridioides difficile* toxin A. GMOS and MOS contain mainly GOS species composed of β1-6- and β1-3-linked galactoses, and 3′- and 6′-sialyllactose. Both GMOS and MOS, combined with lactobacilli, like *Lactobacillus rhamnosus* (LPR, NCC4007), gave synergistic epithelial barrier protection, while no such effect was observed with *Bifidobacterium longum* (BL NCC3001), *Escherichia coli* (Nissle) or fructo-oligosaccharides. Mechanistically, for barrier protection with MOS, (i) viable LPR was required, (ii) acidification of growth medium was not enough, (iii) LPR did not directly neutralize toxin A, and (iv) physical proximity of LPR with the intestinal epithelial cells was necessary. This is the first study, highlighting the importance of structure–function specificity and the necessity of the simultaneous presence of prebiotic, probiotic and host cell interactions required for a biological effect.

## 1. Introduction

Interactions of the gut microbiota with its host modulate numerous physiological functions of the host, including metabolic and immune status [1,2,3,4,5]. Intestinal epithelial cells are at the forefront of this, in mutual alliance with the gut microbial ecosystem. Today, increasing experimental data shows that intestinal epithelial cells are not only a physical barrier, but that these cells play crucial regulatory roles in the defensive systems that are at the interface between the host and the environment [6]. Reciprocal sensing is the hallmark of host-microbiota crosstalk, and its subsequent effects on host health. Several host sensors, such as pattern and danger recognition receptors, and their linked signaling pathways have been elucidated in pioneering studies over the last few decades [7,8]. Equally, microbial compounds have been identified that exert key regulatory functions on the host protection systems [5,9,10].

Increasing data also suggests that microbiota–host misbalance, due to alterations in microbial acquisition and/or composition, can be a root cause of numerous metabolic and inflammatory disease states [11,12]. Classical means of modulating the gut microbiota through consumption of pre- or probiotics have been repeatedly reported to convey health benefits to the host. These include reduced incidence and duration of diarrhea, reduced intestinal inflammation, and maintenance in remission of patients suffering from inflammatory bowel disease (reviewed in [13,14]).

Several studies using various model settings have shown that synbiotics (i.e., the combination of pro- and prebiotic) can be more beneficial to the host than either the pre- or probiotic alone [15,16,17,18,19]. However, this is not universal [20,21], and indicates that research with targeted synbiotics is needed.

*Clostridioides difficile* is an opportunistic pathogen that is the major cause of nosocomial antibiotic-associated diarrhea and pseudomembranous colitis. Pathogenesis involves an alteration of the intestinal microbiota, production of *C. difficile* toxins A and/or B, and inflammation of the colon [22]. Current cures vary from the cessation of antibiotic treatment (for mild cases) to additional antibiotic treatment with metronidazole or vancomycin (for moderate to severe cases) [23,24]. Alternative therapies with probiotics have been tried with *Lactobacillus spp*, with the yeast *Saccharomyces boulardii* being the most successful [25,26,27,28], although not in all populations [28,29]. Prebiotics have also been tested, but further trials will be required before any firm conclusions can be drawn [30,31].

Bovine milk and whey oligosaccharide fractions were identified as potentially interesting prebiotics, as they contain functional oligosaccharides such as sialyllactoses, galactosyllactoses and N-acetylgalactosyllactose [32,33,34]. However, bovine milk contains relatively small amounts of oligosaccharides, making their enrichment for nutritional applications challenging. One route to increasing oligosaccharides in bovine milk derived fractions is to transform part of the lactose in such fractions to galacto-oligosaccharides (GOS). To this end, various β-galactosidases and cell-extracts with β-galactosidase activity have been used [35,36]. A GOS mixture obtained by *Bacillus circulans* β-galactosidase and lactose included numerous di- and oligosaccharides, with β-glycosidic linkages of galactose to C-2, C-3, C-4 and C-6 positions of glucose, galactose, galactose of lactose or galactose of already formed GOS [37,38]. A broad range of oligosaccharide structures was described in GOS mixtures containing at least eight trisaccharides, including four linear (Galβ4Galβ4Glc, Galβ4Galβ3Glc, Galβ4Galβ2Glc and Galβ4Galβ6Glc) and four branched molecules (Galβ6[Galβ2]Glc, Galβ6[Galβ4]Glc, Galβ4[Galβ2]Glc and Galβ6[Galβ3]Glc) [39]. The observation of disaccharides other than lactose further indicates that β-galactosidases can catalyze transglycosylation onto monosaccharides, as well as onto di- and oligosaccharides. Depending on the source of the β-galactosidases, different linkages are formed preferentially [36].

We hypothesized that bovine milk derived oligosaccharide preparations synergize with probiotics. Here, we set out (1) to characterize the main oligosaccharides in bovine whey derived preparations, with and without further enrichment with GOS, and (2) to explore their synbiotic action with *L. rhamnosus* to convey protection of epithelial barrier function against *C. difficile* enterotoxicity, in a simplified in vitro host–epithelial barrier function model system. We found that the bovine milk oligosaccharide-enriched preparation modulated host–bacterial crosstalk, leading to enhanced epithelial barrier function, as measured by paracellular ion flux through transepithelial electrical resistance following *C. difficile* toxin A challenge.

## 2. Materials and Methods

### 2.1. Reagents

All reagents were purchased from Sigma unless otherwise stated. The following oligosaccharides were used: Lactose (Sigma), oligo-fructose (FOS; Orafti P95, Orafti), 3′-sialyllactose (3′SL; Kyowa Hakko Kogyo). *C. difficile* toxin A was purchased from List Biological Laboratories.

### 2.2. Bacterial Strains and Growth Conditions

*L. rhamnosus* (NCC 4007) (LPR), *L. paracasei* (NCC 2461) (ST11) and *B. longum* (BL NCC 3001) were obtained from the Nestlé Culture Collection (NCC). *E. coli* Nissle 1917 (EC) was isolated from Mutaflor (Ardeypharm GmbH). Lactobacilli were grown in Man Rogosa Sharpe (MRS; Difco) broth and EC in brain heart infusion (BHI; Difco) broth overnight at 37 °C. BL NCC3001 was grown in MRS supplemented with 0.05% L-cysteine in an anaerobic chamber with an atmosphere of 5% CO_2_, 10% H_2_ and 85% N_2_ overnight at 37 °C (Scholzen Microbiology Systems, Kriens, Switzerland). Growth was monitored by measurement of the optical density at 600 nm (OD600), and plating to count colony forming units (cfu).

### 2.3. Preparation of Bovine Milk Oligosaccharide-Enriched Fractions

The preparations were derived from bovine milk sweet whey. Briefly, an ultrafiltration permeate of bovine milk whey including oligosaccharides such as sialyllactoses and galactosyl-oligosaccharides (GOS) was concentrated and demineralized, and part of the lactose was removed. The resulting milk oligosaccharide-enriched sample (MOS) was spray dried for storage until further use. Part of the remaining lactose was enzymatically transformed into additional GOS, dubbed here GOS-enriched MOS (GMOS), using an *Aspergillus oryzae* beta-galactosidase (Amano Enzyme, Elgin, IL, USA). For β-galactosidase treatment the spray dried MOS was dissolved at 50% total solids in water at 50 °C. Thereafter, β-galactosidases from *Aspergillus oryzae* was added at 0.375% (w/v) and reacted at 50 °C for 90 min including a heat inactivation step at 85 °C.

To further remove monosaccharides and lactose for functional testing, MOS was dissolved in H_2_O at 30% (w/v) and clarified by passing through an active charcoal bed followed by filtration on a 0.22-µm filter (Millipore, Burlington, MA, USA). The resulting filtrate was loaded onto a preparative Bio-Gel P2 (BioRad, Hercules, CA, USA) column (50 × 850 mm) run with 20 mM ammonium bicarbonate (NH_4_HCO_3_) at a flow rate of 2 mL min^−1^. Fractions containing oligosaccharides and eluting prior to lactose were collected, pooled and lyophilized. This milk oligosaccharide (MOS) preparation contained >80% of the original oligosaccharides, as determined by high performance anion exchange chromatography equipped with pulsed amperometric detection (HPAEC-PAD) profiling, using a CarboPac PA200 analytical column equipped with a CarboPac amino trap column guard (Dionex, Sunnyvale, CA, USA) run on an ICS3000 chromatography system (Dionex). The MOS was virtually salt-free and contained a remaining 1% (w/w) monosaccharides and 4% (w/w) lactose. The overall oligosaccharide enrichment was 16.7 times based on the measured sialyllactoses contents. Sialyllactoses were quantified by HPAEC-PAD as mentioned above using authentic 6′- and 3′-sialyllactoses (Dextra, Reading, UK) as external standards.

### 2.4. Purification of Oligosaccharide Species for Component Isolation and Characterization

Five grams of GMOS was dissolved in 100 mL of water. To this solution 250 mL of cold (+4 °C) ethanol was added and the solution was allowed to stand at +4 °C overnight. The mixture was centrifuged for 5 min at 4000 rpm, and the supernatants were concentrated to about 50 mL with a rotary evaporator. The concentrated solution was applied to a column of successive beds of H+- and Acetate -form ion exchange resins (15 mL each) (AG50W-X8, AG1-X8; BioRad). The eluate was dried, and further purified by passing through an octadecyl-derivatized silica column (BondElut, 5 g; Agilent, Santa Clara, CA, USA). The dried eluate was fractionated by gel permeation chromatography (GPC) in a column of Superdex 30 (5 × 90 cm; Cytiva, GE Health Care, Amersham-Pharmacia, Marlborough, MA, USA) using 50 mM ammonium bicarbonate as the eluent. Absorbance at 214 nm was recorded.

Fractionation by HPAEC (High-pH anion-exchange chromatography) was performed with Dionex 4500i and Dionex DX600 systems (Dionex) using a CarboPac PA-1 column (Dionex). The eluting carbohydrates were detected by pulsed amperometric detector. Two gradients were employed. Gradient 1: Isocratic elution of 45 mM NaOH for 10 min, then a linear gradient from 45 mM NaOH to 150 mM NaOH over 10–30 min, and then a linear gradient from 0 to 200 mM NaAc (sodium acetate) in 150 mM NaOH over 30–60 min. Gradient 2: Linear gradient from 90 mM NaOH to 150 mM NaOH over 0–20 min, and then a linear gradient from 0 to 200 mM NaAc in 150 mM NaOH over 20–60 min. The eluting carbohydrates were manually collected and neutralized by addition of 1 M aqueous acetic acid prior to drying. The glycan samples were desalted by a column of successive cation and anion exchange resins (H+/Ac- forms) and then dried.

### 2.5. Oligosaccharide Characterization

Before Nuclear Magnetic Resonance (NMR) spectroscopy, collected samples were dissolved once in 99.9% deuterium oxide and dried to eliminate excess water. For the NMR analysis, the samples were dissolved in 600 μL of 99.9% deuterium oxide and 1 μL of 1% acetone in 99.9% deuterium oxide was added as an internal standard (2.225 ppm). For the NMR analysis of the trisaccharide components, the samples were twice dissolved in 99.9% deuterium oxide and dried. Samples were then dissolved in 240 μL of 99.996% deuterium oxide and 1 μL of 1% acetone in 99.9% deuterium oxide was added as an internal standard (2.225 ppm). Spectra were collected in Shigemi-NMR-tubes at 296 K using a Varian Unity 600 spectrometer equipped with a cryo-probe.

MALDI-TOF MS (matrix-assisted laser desorption/ionization—time of flight mass spectrometry) was performed on a Bruker Ultraflex TOF/TOF mass spectrometer using a 337-nm nitrogen laser. Samples were dissolved in water to an approximate concentration of 10–20 pmol/μL, and 1 μL of the sample was spotted onto 1.5 μL of recrystallized 2,5-dihydroxybenzoic acid matrix (10 mg/mL in 0.5 mM sodium acetate). External calibration was performed with a malto-oligosaccharide mixture. All measurements were performed in the positive ion reflector delayed-extraction mode, and [M+Na]+ -ions were observed.

For treatment with β-galactosidase, the sample was dissolved in 100 μL of 0.08 M sodium acetate buffer, pH 4.0, containing 10 μg of *A. oryzae* β-galactosidase (Sigma) and incubated for 24 h at 37 °C. The reaction mixture was purified by passing through a small column of successive cation and anion exchange resins (H+/Ac- forms) to remove protein and salts, and finally the sample was subjected to HPAE-chromatography for oligosaccharide isolation.

For treatment with β-N-acetylglucosaminidase, the sample was dissolved in 10 μL of 50 mM sodium acetate buffer, pH 5.5, containing 9 mU of *Streptococcus pneumoniae* β-N-acetylglucosaminidase (Calbiochem, MilliporeMerck, Darmstadt, Germany) and incubated overnight at 37 °C. The reaction mixture was purified by a graphitized carbon column and analyzed by MALDI-TOF MS. A control reaction was done with GalNAcβ1-4GlcNAcβ1-3(Galβ1-4GlcNAcβ1-6)Galβ1-4Glc, which remained intact, indicating that a terminal GalNAcβ- unit is not cleaved by this enzyme.

Linkage analysis was done using periodate oxidation. The sample was dissolved in 50 μL of 50 mM sodium acetate buffer, pH 5.5, containing 8 mM sodium metaperiodate. After incubation overnight at +4 °C, the excess of periodate was destroyed by adding 20 μL of 20 mM aqueous ethylene glycol. The mixture was incubated at room temperature for 30 min, and then 20 μL of 1 M NaBH_4_ in 0.2 M sodium carbonate, pH 10, was added and allowed to react for 2 h at room temperature. The oxidized-reduced glycans were isolated by solid phase extraction on a graphitized carbon column (150 mg; Alltech Grace, Columbia, MD, USA).

### 2.6. Monitoring of Oligosaccharide Profiles

Reducing ends of oligosaccharides were labeled with the fluorescent tag 2-aminobenzamide as previously described [40]. Samples were analyzed using an Ultimate 3000-RS ultra-high-performance liquid chromatography (UHPLC) system equipped with an Acquity BEH Glycan (1.7 mm, 2.1 × 150 mm) and VanGuard BEH amide (1.7 mm, 2.1 × 50 mm) column, both from Waters Corporation (Milford, MA, USA). Compounds were detected after flow was split by an RF-2000 fluorimeter (FLD) and a TriVersa NanoMate^®^ MS interface (Advion, Ithaca, NY, USA) coupled to a QTRAP^®^ 4000 mass spectrometer (Sciex, Framingham, MA, USA).

### 2.7. Cell Culture

T84 cells were obtained from the American Type Culture Collection (ATCC CCL-248) and were maintained in DMEM/F-12 Ham medium containing 5% fetal calf serum (Invitrogen, Waltham, MA; USA), 100 U/mL penicillin, 0.1 mg/mL streptomycin and 2 mM L-glutamine in a humidified 5% CO_2_ atmosphere at 37 °C. Media was replaced three times weekly. For sub-cultures, cells were treated with trypsin-EDTA solution and reseeded. T84 cells were polarized by growth on Transwell inserts (0.4 µm pore; Costar) for 10–14 days. Under these conditions the cells established transepithelial electrical resistance (TEER) of >2000 Ωcm^2^ (Millipore Millicell-ERS Voltohmmeter; MilliporeMerck).

### 2.8. Transepithelial Electrical Resistance Measurements and Cell Integrity

T84 cells on Transwell inserts were cultured overnight in DMEM/F-12 Ham medium containing 2mM L-glutamine. The apical media was replaced with test sample and incubated for 2 h at 37 °C after which *C. difficile* toxin A was added to 10–100 ng/mL. During each experiment duplicate inserts were used for each condition. Variations in toxin specific activity precluded the use of a constant amount. Therefore, concentrations of the toxin were added to T84 cells such that after approximately 20 h of incubation under control conditions only 20% of baseline TEER remained. Experimental T84 responses were normalized to responses in the presence of media alone (0% protection) and MRS at 20% (v/v) (100% protection) as a reference positive control. MRS was previously shown to have protective properties against cell damage caused by toxin A (30). This normalization was chosen due to the variable activity of toxin A batches and stored aliquots. Cell viability was assessed using a cytotoxicity detection kit for the measurement of lactate dehydrogenase released by damaged cells (Roche, Basel, Switzerland). The cell viability test with media alone showed a median mortality of 2% (mean 2.3%) of cells with a minimum and maximum of 1.2% and 3.6%, respectively. Cell mortality with the MRS reference control had a median of 3.5% (mean 3.9%) with a minimum and maximum of 1.8% and 6.9%, respectively. All tested treatment conditions showed mortality in the same range with minimum and maximum from 0% to 6.8%.

### 2.9. Statistics

Data are presented as mean ± SEM and were analyzed by t-test or One-way ANOVA on ranks (Kruskal–Wallis one-way analysis of variance) with Dunn’s multiple comparison test as indicated. *p* < 0.05 was considered significant.

## 3. Results

### 3.1. Structural Characterization of Oligosaccharides in MOS and GMOS Preparations

**Figure 1:**
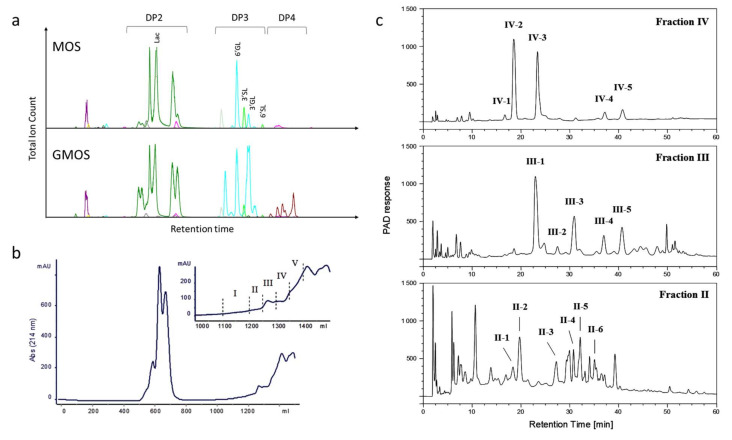
(**a**) Illustration of LC-MS traces of MOS and GMOS; (**b**) Gel-filtration chromatography of GMOS indicating isolated fractions. The eluted carbohydrates were pooled into five fractions for subsequent analyses as shown in the inset; (**c**) HPAE chromatography of gel-filtration chromatography fractions II–IV. Fractions IV and III were run with gradient 1, and fraction II with gradient 2. The indicated fractions were analyzed as described in the text. Lac, lactose; 3′GL, 3′Galactosyllactose; 6′GL, 6′Galactosyllactose; 3′SL, 3′Sialyllactose; 6′SL, 6′Sialylactose; DP, degree of polymerization.

Comparison of MOS and GMOS by liquid chromatography with mass spectrometry (Figure 1A) illustrates that the materials are composed predominantly of di-, tri and tetrasaccharides (DP2, DP3 and DP4), with a marked change in the proportion of the different oligosaccharides between MOS and GMOS. From m/z ratios and retention time comparison to authentic standards, we identified lactose, 3′- and 6′sialyllactose (3′SL, 6′SL), as well as 3′- and 6′galactosyllactose (3′GL, 6′GL), in MOS and GMOS (Figure 1A). Their presence was confirmed by subsequent detailed characterization of GMOS.

The major oligosaccharides in GMOS were structurally characterized using fractionation, isolation and characterization by mass spectrometry and NMR. By size exclusion chromatography, GMOS was divided into five fractions (Figure 1B). Fractions II, III and IV were further fractionated to isolate and characterize individual components, as illustrated (Figure 1C). The 1H-NMR assignments of the isolated components are shown in Supplementary Materials Tables S1–S3 and Figures S1–S3. We characterized 12 oligosaccharide structures from the isolated fractions II, III and IV (Table 1). A detailed description of the structure identification for each fraction is provided in Appendix A.

Briefly, fraction IV-1 was identified as Galβ6Gal and fraction IV-2 was tentatively identified as lactose. The IV-3 and IV-4 components were identified as (Hex)3 trisaccharides, namely Galβ6Galβ4Glc and Galβ3Galβ3Glc, respectively. Another (Hex)3 was seen in component IV-5, namely, Galβ3Galβ4Glc. The III-1 fraction contained a mixture of (Hex)3 and (HexNAc)1(Hex)2 species that were identified to be Galβ6Galβ4Glc and GalNAcα3Galβ4Glc. Fraction III-2 was identified as the (Hex)4 tetrasaccharide Galβ6Galβ6Galβ6Glc and fraction III-3 Galβ6Galβ6Galβ4Glc. Fractions III-4 and IV-4 were identical (Galβ3Galβ3Glc), as were fractions III-5 and IV-5 (Galβ3Galβ4Glc). Another tetrasaccharide containing a HexNAc was seen in fraction II-1 and identified to be the structure Galβ6GalNAcα3Galβ4Glc. The structures in fractions II-2 and III-3 were identical (Galβ6Galβ6Galβ4Glc), and fraction II-3 was identical to that of fraction IV-5 Galβ3Galβ4Glc.

The fractions II-4, II-5 and II-6 were identified as additional tetrasaccharides, Galβ3Galβ6Galβ4Glc, Galβ6Galβ3Galβ4Glc and Galβ3Galβ3Galβ4Glc, respectively.

### 3.2. Both MOS and GMOS Synergize with LPR

We chose to functionally characterize the MOS and GMOS preparations as synbiotic with *Lactobacillus rhamnosus* LPR, using a simplified in vitro host epithelial barrier function model system that employs the barrier disruptive properties of *C. difficile* toxin A.

**Figure 2:**
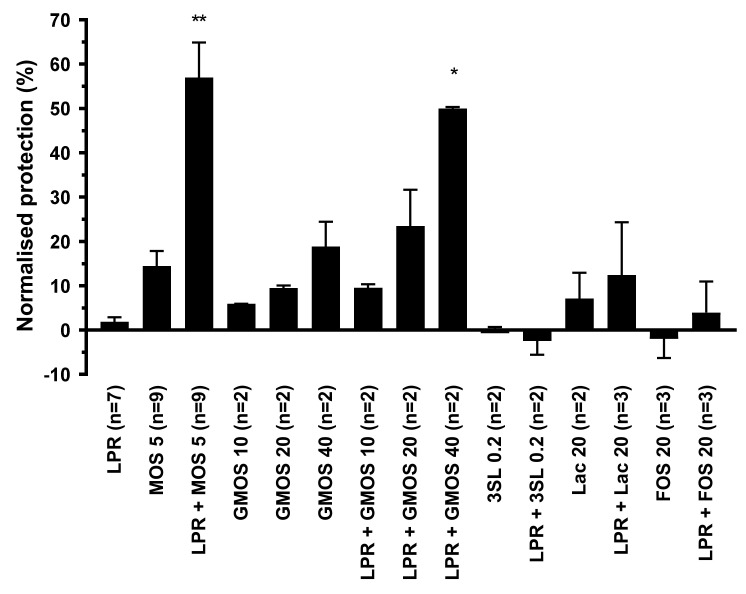
Protection of T84 epithelial cells from barrier integrity damage by *C. difficile* toxin A. T84 cells were preincubated for 2 h with MOS, GMOS, Lactose (Lac) or 3′Sialyllactose (3′SL) in the absence or presence of 10^5^/mL *L. rhamnosus* (LPR) prior to the addition of *C. difficile* toxin A. T84 transepithelial resistance was measured before sample addition and after 20 h in the presence of toxin A. Protection of resistance was normalized to that of T84 in media alone (0%) and MRS (100%). Data are expressed as mean ± SEM, number of independent experiments with *n* = 2 replicates each are indicated in the figure legend (*n* = 2–9). Statistical difference to LPR alone are indicated (One-way ANOVA on ranks with Dunn’s multiple comparison test) * *p* < 0.05, ** *p* < 0.01.

Polarized T84 cells were exposed on the apical surface to either LPR alone, MOS or GMOS alone, or a combination of 5 mg/mL MOS or 10 to 40 mg/mL GMOS with LPR at 10^5^ cfu/mL. As controls, we used lactose and 3′sialyllactose (3′SL), both present in MOS and GMOS, with and without LPR. Subsequently, cells were challenged with *C. difficile* toxin A, and barrier disruption was assessed through trans-epithelial resistance measurement. LPR alone at 10^5^ cfu/mL did not show any barrier protection, while MOS and GMOS alone showed a minor dose-dependent effect (Figure 2). In combination with LPR, both MOS and GMOS, pre-incubated for 2 h, showed considerable barrier protective effects.

Lactose is the core disaccharide (Galβ4Glc) at the base of most of the structures identified in MOS and GMOS, hence we included lactose in our functional tests. Lactose at a concentration as high as 20 mg/mL, either alone or in the presence of LPR, did not protect. When 0.2 mg/mL 3′SL (the approximate concentration present in MOS at 5 mg/mL) was tested in the absence or presence of LPR, no protection was seen. However, at the much higher concentration of 10 mg/mL, 3′SL in combination with LPR gave significant protection (data not shown).

To benchmark our findings against a classic prebiotic, we chose to use a structurally distinct oligosaccharide preparation. Prebiotic Fructo-oligosaccharides (FOS), which are elongations of sucrose by fructose units and structurally very different from MOS, did not show any protection, either alone or in combination with LPR.

Since we saw similar biological activity between MOS and GMOS, we decided to focus on MOS for a more detailed investigation of the observed activity.

### 3.3. LPR and MOS Synergize to Protect Against Toxin A

**Figure 3:**
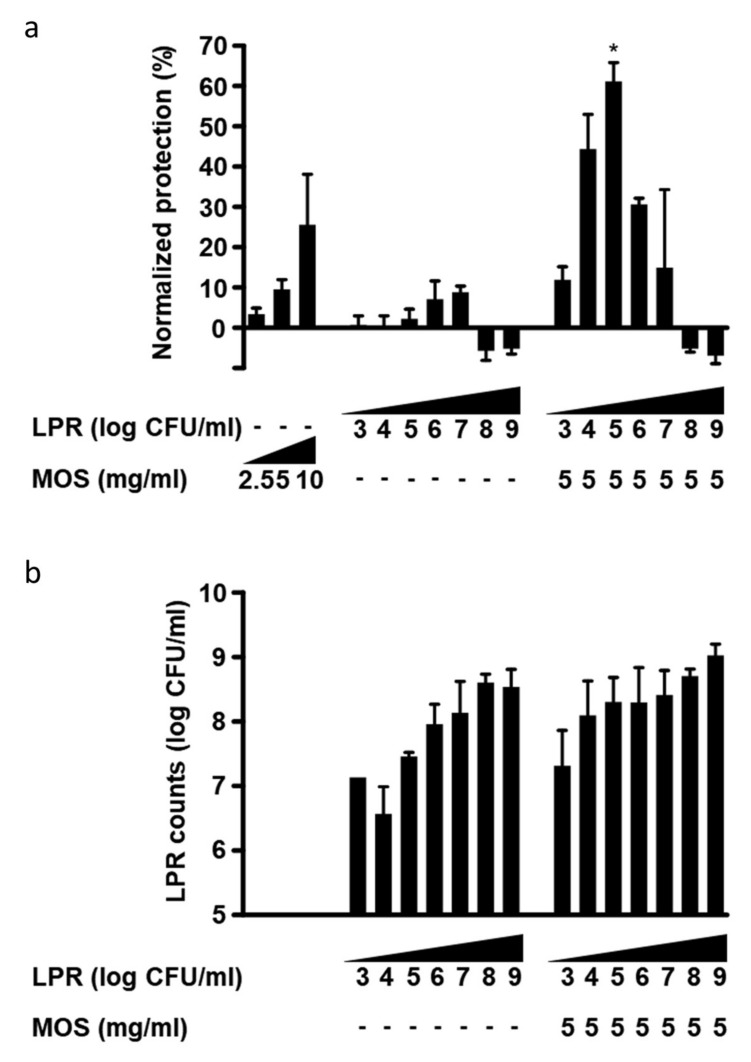
(**a**) Protection of T84 epithelial cells from barrier integrity damage by *C. difficile* toxin A. T84 cells were preincubated for 2 h with MOS, in the absence or presence of 10^3^ to 10^9^/mL *L. rhamnosus* (LPR) prior to the addition of *C.difficile* toxin A. T84 transepithelial resistance was measured before sample addition and after 20 h in the presence of toxin A. Protection of resistance was normalized to that of T84 in media alone (0%) and MRS (100%). Data are expressed as mean ± SEM, *n* = 3 independent experiments, except LPR at 10^4^, 10^6^, 10^8^/mL (*n* = 2), independent experiments with *n* = 2 replicates each. Statistical difference from LPR alone plus MOS alone is indicated * *p* < 0.05. (**b**) Concentration of LPR after incubation for 20 h on T84 cells in absence or presence of 5 mg/mL MOS and at different initial inoculation concentrations from 10^3^ to 10^9^ cfu/mL. Data are expressed as mean ± SEM, *n* ≥ 2 independent experiments with *n* = 2 replicates each. Statistical difference to corresponding LPR concentration without MOS by unpaired t-test is indicated * *p* < 0.05.

We exposed polarized T84 on the apical surface to serial dilutions of either LPRor MOS alone, or a combination of 5 mg/mL MOS with serial dilutions of LPR. Subsequently, cells were challenged with *C. difficile* toxin A. MOS alone was able to confer partial protection in a concentration-dependent manner (Figure 3a). LPR alone also conferred a minor, yet concentration-dependent protection reaching, a maximum of only 10% normalized protection with 10^7^ cfu/mL (Figure 3a). At bacterial concentrations greater than 10^7^ cfu/mL, the apparent effect was reversed. LPR grew in the presence of T84 cells with saturation at approximately 10^9^ cfu/mL (Figure 3b).

In order to further characterize the protective effect of the synbiotic combination of LPR and MOS against toxin A, a MOS concentration of 5 mg/mL, which gave less than 10% normalized protection, was chosen and co-incubated with serial dilutions of LPR. Interestingly, with a constant amount of MOS, we observed a bacterial concentration-dependent increase in protection. A maximum and synergistic protection was obtained with a starting LPR concentration of 10^5^ cfu/mL (Figure 3a). Considerable protection was also seen when 10-fold less or 10-fold more LPR was used, but at initial LPR concentrations of >10^7^, no protection was observed.

The presence of MOS and inoculation of LPR levels above 10^3^ cfu/mL led to final LPR concentrations between 10^8^ and 10^9^ cfu/mL (Figure 3b). Comparable final bacterial densities were seen without MOS when starting inocula were above 10^6^ cfu/mL. With low starting concentrations of LPR (10^3^–10^6^ cfu/mL), bacterial growth was greater in the presence than in the absence of MOS, suggesting the growth-promoting activity of MOS (Figure 3b). When present alone, these very high bacterial loads were not deleterious to monolayer permeability, as in the absence of toxin A, T84 cells maintained high TEER values (data not shown). In the presence of toxin A, LPR inocula above 10^7^ cfu/mL resulted in substantial TEER reduction.

### 3.4. Specificity of Probiotics

**Figure 4:**
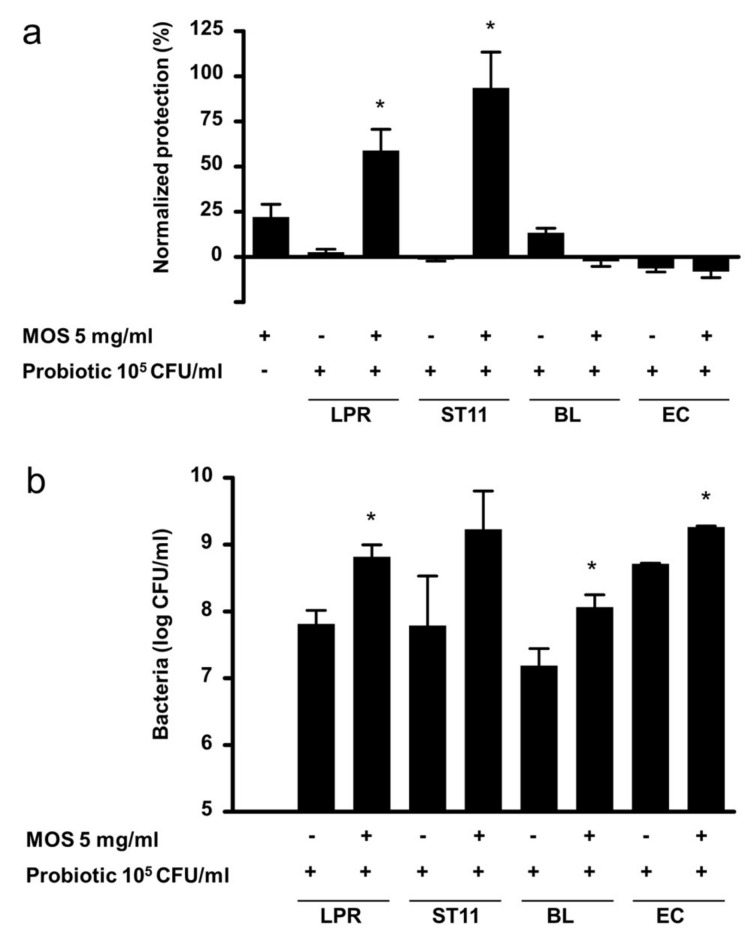
(**a**) Protection of T84 epithelial cells from barrier integrity damage by *C. difficile* toxin A. T84 cells were preincubated for 2 h with MOS, in the absence or presence of 10^5^/mL *L. rhamnosus* (LPR), *L. paracasei* (ST11), *B. longum* BL NCC3001 (BL) or *E. coli* Nissle (EC) prior to the addition of *C.difficile* toxin A. T84 transepithelial resistance was measured before sample addition and after 20 h in the presence of toxin A. Protection of resistance was normalized to that of T84 in media alone (0%) and MRS (100%). Data are expressed as mean ± SEM, *n* ≥ 2 independent experiments with *n* = 2 replicates each. Statistical difference by *t*-test to the probiotic alone plus MOS alone is indicated * *p* < 0.05. (**b**) Concentration of LPR, ST11, BL NCC3001 and EC after incubation for 20 h on T84 cells in the absence or presence of 5 mg/mL MOS. Data are expressed as mean ± SEM, *n* = 5–8 except BL (*n* = 3) and EC (*n* = 2) independent experiments with *n* = 2 replicates each. Statistical difference by *t*-test to corresponding probiotic concentration without MOS is indicated * *p* < 0.05.

To assess whether the synergistic protection observed with LPR and MOS was unique to LPR, we tested other known probiotic bacteria from different taxonomic groups. By themselves, none of the probiotics tested conferred protection against toxin A (Figure 4a). In the presence of 5 mg/mL MOS *Lactobacillus paracasei*, ST11 gave significant and synergistic protection of a similar magnitude to that seen with LPR. On the other hand, neither *Bifidobacterium longum* BL NCC3001 nor the Gram-negative probiotic *Escherichia coli* Nissle (EC) showed any protection. The two lactobacilli and *E. coli* Nissle grew to similar final cell densities in the absence of MOS (Figure 4b). *Bifidobacterium longum* BL NCC3001 grew to about 10 times lower levels. In the presence of MOS, all probiotics grew to roughly 10 times higher final cell densities, indicating that MOS has bacterial growth-promoting activity. However, only the combination of MOS with lactobacilli provided protection.

### 3.5. Characteristics of the Requirements for Protection

**Figure 5:**
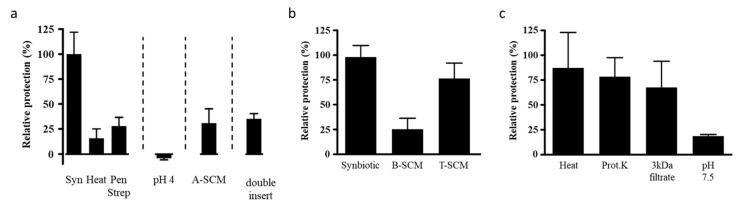
(**a**) T84 cells were preincubated with MOS and either live *L. rhamnosus* (LPR) (synbiotic, Syn), heat-treated LPR (Heat) or live LPR and penicillin and streptomycin (Pen Strep) for 2 h prior to the addition of *C. difficile* toxin A. T84 cells were preincubated with media adjusted to pH 4 for 2 h prior to the addition of toxin A (pH 4). T84 cells were incubated with the filter sterilized spent culture media prepared from LPR grown overnight with MOS and toxin A (A-SCM). T84 cells were preincubated with MOS and LPR, with LPR in a second smaller insert placed above the primary insert and thus physically separated from the T84 cells, for 2 h prior to the addition of toxin A (double insert). In all T84 transepithelial resistance was measured before sample addition and 20 h following toxin A. (**b**) T84 cells were preincubated with LPR and MOS (Synbiotic), the filter sterilized overnight spent culture supernatant of LPR and MOS (B-SCM), or the filter sterilized spent culture supernatant of LPR and MOS in the presence of T84 cells (T-SCM) for 2 h prior to the addition of *C. difficile* toxin A. (**c**) The T-SCM was boiled (Heat), digested with proteinase K (Prot.K), filtered through a 3 kDa cutoff filter (3 kDa filtrate) or adjusted to pH 7.5 prior to preincubation on T84 cells. Following a 2 h incubation toxin A was added. T84 transepithelial resistance was measured before sample addition and after 20 h in the presence of toxin A. Protection of the resistance was determined relative to that of the synbiotic and results are expressed as mean ± SEM with *n* = 2 for heat and *n* = 3 for all other conditions.

First, we tested whether viable LPR was necessary in order to exert its positive effect with MOS. Neither the combination of MOS with heat-inactivated LPR, nor of MOS with LPR incubated in the presence of the antibiotics penicillin and streptomycin, gave protection, compared to the synbiotic of MOS with live LPR (Figure 5a). Thus, viable LPR is required for protection.

LPR, like other probiotics, is a lactic acid-producing bacterium, and acidification of the growth milieu has been proposed as one of the mechanisms of protective probiotic activity within the gut. LPR in the absence of MOS slightly acidified the growth media (pH 6.8 ± 0.5 vs. media alone pH 7.3 ± 0.2) whereas the synbiotic significantly acidified (pH 4.9 ± 0.3) the media. To directly test whether the acidity of the medium as such provided protection against toxin A, we acidified the apical chamber with lactic acid prior to adding toxin A. Neither at pH 5 nor pH 4 was protection observed (Figure 5a). It should be noted that *Bifidobacterium longum* BL NCC3001 combined with MOS acidified the medium similarly to LPR (data not shown), but did not provide protection. Thus, acidification as such is unlikely to be the mechanism through which protection of the host cell was achieved.

Another proposed mechanism of action of the probiotics is the enzymatic neutralization of toxic products. To test whether LPR could directly neutralize the toxin, we incubated toxin A overnight in the presence of LPR and MOS. After removal of LPR by filter sterilization, the spent culture media containing any remaining MOS and toxin A (A-SCM) still caused significant reduction of T84 TEER, similar to control conditions with freshly added toxin A, suggesting that toxin A was still present and active (Figure 5a). Therefore, the LPR MOS synbiotic did not directly neutralize toxin A.

In another possible mechanism, probiotic bacteria have been suggested to exert their activity through binding to epithelial cells. This binding could sterically hinder toxin A’s binding to its receptor, and/or activate other unidentified signaling pathways. Under the conditions tested here, adhesion of LPR to T84 cells did not appear to be modified by the presence of MOS (data not shown). Thus, the observed protection by the LPR with MOS is unlikely to be due to the steric hindrance of toxin A binding to host cells, or increased LPR host cell communication via increased adhesion.

As adhesion did not seem to change, we next sought to determine if LPR adhesion was at all important for activity. To test this, LPR and MOS were incubated on T84 cells, with the LPR and T84 cells kept physically separated by an additional Transwell insert. When challenging this setup with toxin A, no protection was seen (Figure 5a). Consistent with this finding, the spent culture medium, obtained from MOS prepared in cell culture medium and fermented by LPR (B-SCM) in the absence of T84 cells, was not enough to protect naïve T84 cells against toxin A (Figure 5b). Thus, to get protection, the three partners LPR, MOS and T84 cells had to be present together, with the necessity of the physical contact or proximity of LPR and epithelial cell. This suggests MOS stimulated crosstalk between the probiotic LPR and the T84 host cells in order for protection to occur.

We next tested whether soluble factors produced during this crosstalk could confer protection to naïve T84 cells. As mentioned above, the B-SCM from the LPR MOS fermentation alone without the host cells was not protective (Figure 5b). However, filter sterilized conditioned media from the overnight incubation of the tripartite mix of LPR with MOS on T84 cells (T-SCM) protected naïve T84 cells, comparable to the control synbiotic samples (Figure 5b). The control SCM from the overnight incubation of the mix of either LPR and T84 (D1-SCM) or MOS and T84 (D2-SCM) did not confer protection (data not shown).

To explore the nature of the generated protective factor, we treated T-SCM with heat and Proteinase K, and filtered the fraction through a nominal 3 kDa molecular weight cut-off filter, and neutralized T-SCM to pH 7.5 (Figure 5c). The protective factor passed the 3 kDa filtration, and was insensitive to heat and Proteinase K, but sensitive to pH adjustment.

## 4. Discussion

Reciprocal host–microbe interactions through metabolites and direct physical interactions are the hallmark of the gut ecosystem. Aberrations of host–microbe interactions are increasingly recognized to have causal relations to the host physiology. To strengthen the gut ecosystem, probiotics and prebiotics, or their combinations, are a promising option. Important structure and function relations may exist, requiring an educated pairing of pro- and prebiotics. Here, we characterized bovine milk derived oligosaccharide preparations from a structural and functional perspective, in combination with probiotics of different taxonomic groups. We found an interesting synergy specifically with lactobacilli, which required a tripartite interaction and crosstalk between the lactobacilli and host epithelial cells, stimulated by the bovine milk derived oligosaccharide preparations MOS and GMOS.

Our study shows the structural characterization of 11 major galactooligosaccharide components in GMOS. We characterized one disaccharide (Galβ6Gal) and four trisaccharides [Galβ3Galβ4Glc (3′GL), Galβ6Galβ4Glc (6′GL), Galβ3Galβ3Glc, and GalNAcα3Galβ4Glc]. Additionally, we characterized six tetrasaccharide components: Galβ3Galβ3Galβ4Glc, Galβ6Galβ3Galβ4Glc, Galβ3Galβ6Galβ4Glc, Galβ6Galβ6Galβ4Glc, Galβ6Galβ6Galβ6Glc and Galβ6GalNAcα3Galβ4Glc. Several of these structures, especially the trisaccharides 3′GL and 6′GL, were previously reported in commercial GOS preparations (for example, Vivinal-GOS) [39]. These trisaccharides are the main structures naturally present in MOS, together with several disaccharides and only traces of tetrasaccharides. With the β-galactosidase treatment, we increased di-, tri- and tetrasaccharides as compared to the untreated MOS preparation. Di-, tri- and tetrasaccharides, and larger components, have been reported previously following trans-glycosylation reactions, but few structures have been characterized in detail. Of the tetrasaccharides characterized here, Galβ3Galβ3Galβ4Glc [41] and Galβ3Galβ6Galβ4Glc [42] have been previously described. In our ingredient preparations, we observed primarily β-3- and β-6- linked galactoses, whereas another well-characterized GOS, Vivinal GOS, is composed to a larger extent of β-4-linked galactoses [39]. Some oligosaccharides that we characterized do not have a terminal lactose (Galβ4Glc), but terminate with Galβ3Glc or Galβ6Glc. This indicates that the enzyme used for trans-glycosylation can also use free glucose as an acceptor substrate. Similarly, many oligosaccharide species in Vivinal-GOS terminate with Galβ3Glc, Galβ6Glc or Galβ2Glc [39]. Interestingly, in Vivinal GOS several branched oligosaccharide species have been identified with galactose attached in more than one position of the terminal glucose. While we did not observe such structures in our preparations, it is possible that they exist at low concentrations and were not isolated.

Numerous studies show that GOS are generally bifidogenic [43,44,45,46,47,48]. However, the effect of the structure of GOS, such as its linkages and size, is poorly established. *B. circulans* β-galactosidase-produced Vivinal-GOS was fermented by four different *Bifidobacterium* species, and the remaining oligosaccharides were profiled by mass spectrometry. Interestingly, each bacterium showed a different capacity to utilize GOS [49]. While *B. longum* subsp. *infantis* (ATCC 15697) utilized all GOS available, *B. longum* subsp. *longum* (DJO10A) grew only very slowly on GOS as the sole carbon-source, and *B. adolescentis* (ATCC 15703) seemed to preferentially consume smaller sized oligosaccharides. GOS produced by β-galactosidase from *Bifidobacterium bifidum* is composed primarily of β1,6- and β1,3-linked GOS [41], and was shown to be preferentially utilized by *B. bifidum* over other known prebiotic oligosaccharides including Vivinal-GOS [50].

The gut microbiota balancing effect of GOS goes beyond a bifidogenic effect, in that GOS is also reported, for example, to reduce the incidence and symptoms of travelers’ diarrhea [43]. Little is known about the structural requirements favored, but in vitro adhesion experiments indicate that for specific enteric pathogens, specific GOS structures could be responsible for the protective effect of GOS [51]. Here, we observed growth-promotion of *B. longum* BL NCC3001 with MOS, yet we did not observe protection from toxin A. In a clinical trial, GMOS feeding also strongly increased bifidobacteria in infant stool, in particular *B. longum*. Interestingly, although only numerical, GMOS also increased the relative abundance of *Lactobacillus* [46].

In our study, we show in vitro synergistic protection against the epithelial barrier disruptive effects of *C. difficile* toxin A by the synbiotic mixture of MOS or GMOS, and specifically either *L. rhamnosus* NCC 4007 (LPR) or *L. paracasei* NCC 2461. These results support the notion that synbiotic-use can provide greater benefits (either additive or synergistic) than the simple use of either probiotic or prebiotic alone. We did not observe protection when we combined MOS with probiotics from other taxonomic groups, or when we used LPR with FOS, another class of prebiotic. Our results therefore suggest that a careful evaluation of specific combinations of pro- and prebiotic is required, and that the mechanism for generating protection may be unique to each combination.

It is noteworthy thar protection is brought about by MOS-stimulated crosstalk between the host epithelial cells and LPR. This crosstalk leads to the liberation of a small protease- and heat-resistant compound(s), which on its own could confer epithelial barrier protection in naïve host cells that had previously seen neither the bacteria nor MOS. This suggests that the MOS-stimulated bacterial host cell crosstalk could have protective effects beyond the immediate site of interaction.

How might this synbiotic stimulate epithelial cell resistance to toxin A? Toxin A’s effects on host epithelial cells are well described, and lead to two main barrier disruptive outcomes, namely, a cytopathic effect seen in cytoskeleton breakdown and loss of tight cell–cell contact, and a cytotoxic effect, leading to activation of inflammatory processes followed by programmed cell death [52]. The conditioned media experiments indicate that part of the activity resides in a soluble factor(s) released from the bacteria, the T84 host cells, or both. In addition, the release of the protective factor(s) requires MOS-stimulated crosstalk between host cells and bacteria, mediated by close contact. Tao et al. have shown in vitro that soluble factors from *L. rhamnosus* GG (LGG) spent culture media activate MAP kinases, and induce expression of the heat shock proteins Hsp25 and Hsp72 in intestinal epithelial cells [53]. Indeed, inducible cytoprotective Hsp72 has been shown to be important for protection against toxin A [54]. In T84 cells, however, this is not likely to be the principal mechanism involved. First, these cells already express high amounts of Hsp72 under non-stressed conditions ([55] and P.I.Duncan, unpublished results). Second, production of the LGG bioactive factors necessary for Hsp induction requires growth in MRS, with growth in RPMI tissue culture media being ineffective (our cells are grown in DMEM/F12 tissue culture media). Third, the LPR+MOS+T84-conditioned media is insensitive to proteinase K treatment, in contrast to that seen by Tao et al. [53].

Yan and Polk have similarly shown that LGG or its soluble factors recovered from spent MRS growth media or RPMI cell culture media can stimulate the anti-apoptotic kinase Akt [56,57], but in contrast to the results of Tao et al., MAP kinases were not stimulated [53]. Thus, the spectrum of bioactive factors produced by *L. rhamnosus* is diverse, and likely dependent on their manner of stimulation [58]. Further, a soluble bioactive factor(s) released from the yeast probiotic *Saccharomyces boulardii* (grown in RPMI) has been shown to block toxin A in vitro and in vivo [59]. This factor blocks toxin A-induced MAP kinases and IL-8 production.

We have previously shown that MRS growth medium itself is a potent inhibitor of toxin A, both in vitro and in vivo [60]. It is currently unclear if the LPR+MOS synbiotic functions in a mechanistically similar manner to MRS in protection against toxin A in T84 cells.

Our results suggest that the synbiotic blend does not directly neutralize toxin A. Rather, LPR in the presence of MOS and T84 epithelial cells leads to the induction of a bioactive factor(s) that then brings about protection of T84 host cells. The necessity for LPR and T84 being in proximity to one another suggests that adhesion of LPR to T84 cells is required, and that signal transduction is occurring. However, gross T84–bacterial adhesion was not altered by MOS. Further experimentation will be required to determine if and how MOS alters the quality of the interaction.

An Hsp90 family member, dubbed glycoprotein 96 (gp96), has been identified to be a human toxin A receptor [61]. Gp96 is involved in numerous innate and adaptive immune functions, being required both for intra- and extracellular expression of TLRs [62]. In Caco2 epithelial cells, expression of gp96 was reported to increase upon interaction of the cells with *Lactobacillus fermentum* [63]. To confer protection against toxin A, we may speculate that an LPR+MOS+T84-induced factor alters signaling through gp96. This could be by reducing surface expression of gp96, or through allosteric regulation of this receptor. Future studies are needed to elucidate the mechanism of action, and to identify which components in MOS and GMOS are required for the observed synergy with lactobacilli to confer protection.

*Lactobacillus rhamnosus* and other *Lactobacillus* species govern host interactions and inflammation-related pathways through multiple factors [64]. These include adhesive pili or fimbriae, lipoteichoic acid molecules, major secreted proteins and galactose-rich exopolysaccharides, as well as specific CpG DNA motifs. Taken together, with current limited insight, we speculate that the yet-unidentified soluble component liberated upon MOS-stimulated LPR–host cell interaction likely reduced the effect of toxin A through the alleviation of the inflammatory pathway triggered by toxin A.

In summary, our structural and functional characterizations demonstrate the potential of synbiotics to deliver bona fide synergistic effects. The protection against the barrier disruptive effect, seen as increased paracellular ion flux, of toxin A was greater with the synbiotic mix MOS-LPR than it was with the addition of the responses of MOS and LPR individually. Significantly, this is the first study, to the best of our knowledge, highlighting the necessity of the simultaneous presence of prebiotic, probiotic and host cell for productive cross-talk targeting increased epithelial barrier function.

## Figures and Tables

**Table 1:** Identified oligosaccharide structures.

Collected Fraction	Identified Structure
IV-2	Galβ4Glc
IV-1	Galβ6Gal
IV-3/III-1	Galβ6Galβ4Glc
IV-4/III-4	Galβ3Galβ3Glc
IV-5/III-5/II-3	Galβ3Galβ4Glc
III-1b	GalNAcα3Galβ4Glc
III-3/II-2	Galβ6Galβ6Galβ4Glc
II-4	Galβ3Galβ6Galβ4Glc
II-5	Galβ6Galβ3Galβ4Glc
II-6	Galβ3Galβ3Galβ4Glc
II-1	Galβ6GalNAcα3Galβ4Glc
III-2	Galβ6Galβ6Galβ6Glc

## Supplementary Materials

The following are available online at https://www.mdpi.com/2072-6643/12/7/2007/s1, Table S1: Proton chemical shift values (ppm) of GOS disaccharide and trisaccharide components., Table S2: Proton chemical shift values (ppm) of GOS tetrasaccharide components., Table S3: Proton chemical shift values (ppm) of GOS tetrasaccharide components., Figure S1: 1H-NMR spectra of GOS components from fraction IV. (A) IV-1, (B) IV-3, (C) IV-4 and (D) IV-5. See text and Table S1 for assignments., Figure S2: 1H-NMR spectra of GOS components from fraction III. (A) III-1b, (B) III-2 and (C) III-3. See text and Tables S1–S3 for assignments., Figure S2: 1H-NMR spectra of GOS components from fraction II. (A) II-1, (B) II-4, (C) II-5 and (D) II-6. See text and Tables S2 and S3 for assignments.

## Appendix A. Supplementary Description of Structure Identification for Each Fraction

The fraction IV-1 was identified as a (Hex)2 by MALDI-TOF MS (m/z 365.1, [M+Na]+) (not shown), and the 1H-NMR spectrum (Figure S1A) is identical to that of authentic disaccharide Galβ6Gal. The spectrum reveals typical anomeric signals of a reducing galactose unit (H-1α 5.265 ppm, H-1β 4.594 ppm). Two signals with a typical β-glycosidic coupling (7.5 Hz) are observed at 4.444 and 4.454 ppm. Spectral integration showed that these two are equivalent to one proton, and thus these represent the terminal β1,6-linked Gal-unit H-1 α- and β-anomeric signals.

The fraction IV-2 tentatively identified as lactose by its elution time was confirmed by 1H-NMR analysis (spectrum not shown).

The IV-3 component was identified as a (Hex)3 by MALDI-TOF MS (m/z 527.1, [M+Na]+) (not shown). The 1H-NMR spectrum (Figure S1B) shows H-1 signals from three monosaccharide units, and is identical to that of the trisaccharide Galβ6Galβ4Glc (REF 6 Urashima 1994). The reducing end glucose signals are similar to those in lactose (H1α 5.222 ppm, H1β 4.669 ppm), and the H-1 signal of the Galβ1-4 unit has experienced a small downfield shift from 4.452 to 4.461 ppm. The H-1 of the terminal Galβ1-6 unit resonates at 4.485 ppm. In addition, a characteristic H-6 signal of a 6-substituted Gal unit is seen at 4.07 ppm (1H by integration).

The IV-4 component was identified as a (Hex)3 species by MALDI-TOF MS (m/z 527.1, [M+Na]+) (not shown), and the 1H-NMR spectrum (Figure S1C) shows great similarity to that of the trisaccharide Galβ3Galβ4Glc (REF 6 Urashima 1994), except for the H-1 signal of the penultimate Gal-unit (4.484 ppm). The spectrum shows typical reducing end Glc-unit signals (H1α 5.222 ppm, H1β 4.663 ppm), and a characteristic H-1 signal for a terminal Galβ1-3 unit (4.602 ppm). This spectrum also contains a typical H-4 signal of a 3-substituted Gal-unit at 4.194 ppm. Therefore, the probable sequence is Galβ1-3Galβ1-Glc, where the linkage to the reducing Glc is either β1-6, β1-3 or β1-2. Both β1-6 and β1-2 linkages can be excluded as H-6 signal of the reducing Glc-unit would reside around 4.21 to 4.29 ppm if the Glc was 6-substituted (7). The H-1 signals of β1-2-substituted Glc-units are very characteristic (e.g., in Glcβ1-2Glc: H-1α 5.453 ppm and H-1β 4.794 ppm). Therefore, we propose that the fraction IV-4 represents Galβ3Galβ3Glc.

The IV-5 component was identified as a (Hex)3 species by MALDI-TOF MS (m/z 527.1, [M+Na]+) (not shown). The anomeric proton region of fraction IV-5’s 1H-NMR spectrum (Figure S1D) is practically identical to that of the trisaccharide Galβ3Galβ4Glc (6). The spectrum shows typical reducing end Glc-unit signals (H1α 5.224 ppm, H1β 4.666 ppm). Compared to lactose NMR signals, the H-1 signal of the Galβ1-4 unit has experienced a distinctive downfield shift from 4.452 to 4.511 ppm. The H-1 of the terminal Galβ1-3 unit is observed at 4.614 ppm. Moreover, the spectrum contains a typical H-4 signal of a 3-substituted Gal-unit at 4.197 ppm.

The III-1 fraction was identified as a mixture of (Hex)3 and (HexNAc)1(Hex)2 species by MALDI-TOF MS (m/z 527.1 and m/z 568.2, respectively) (not shown). The 1H-NMR analysis (not shown) showed that the major component was Galβ6Galβ4Glc. The minor component contained an N-acetyl group, as revealed by the acetyl proton signals at 2.045 ppm. To purify the minor component, an aliquot of the fraction III-1 was subjected to A. oryzae β-galactosidase treatment. The hydrolyzate was then subjected to HPAEC, which showed peaks of Gal and Glc (co-eluting in this gradient) and lactose, and a small peak at the original fraction III-1 elution position. This β-galactosidase-resistant material (fraction III-1b) was analyzed by proton NMR (Figure S2A). The spectrum showed typical signals for a β1-4-substituted reducing end Glc-unit (H-1α 5.225 ppm, H-1β 4.667 ppm), and a Galβ1-4 H-1 signal at 4.514 ppm. Moreover, a characteristic H-4 signal of a 3-substituted Gal unit is seen at 4.111 ppm. Thus, the reducing end disaccharide was identified as 3-substituted lactose. The terminal unit is an α-glycosidically linked HexNAc unit, as revealed by the H-1 signal at 5.082 ppm (J 3.5 Hz). This unit was identified as α-GalNAc by the characteristic H-2 signal at 4.231 ppm and the H-5 signal at 4.195 ppm [Wieruszeski, 1990]. Taken together, the minor component of fraction III-1 was identified as GalNAcα3Galβ4Glc.

The fraction III-2 was identified as a (Hex)4 tetrasaccharide by MALDI-TOF mass spectrometry (m/z 689.2, [M+Na]+) (not shown). The 1H-NMR analysis shows reducing end Glc-unit H-1α at 5.223 ppm and H-1β at 4.684 ppm (Figure S2B). The H-1β value implies that this component does not contain lactose as the reducing end unit. This notion is supported by the two H-6 signals at 4.213 and 4.287 ppm, which are assigned to 6-substituted Glc residue [Calis, 2002]. The reducing end disaccharide is Galβ6Glc. In addition, a characteristic H-6 signal of a 6-substituted Gal unit is seen at 4.07 ppm (2H by integration). This confirms the presence of two 6-substituted galactose residues. Thus, the fraction III-2 tetrasaccharide was identified as Galβ6Galβ6Galβ6Glc.

The fraction III-3 was identified as a (Hex)4 tetrasaccharide by MALDI-TOF mass spectrometry (m/z 689.2, [M+Na]+) (not shown). The fraction III-3 1H-NMR spectrum shows reducing end Glc-unit signals H-1α at 5.222 ppm and H-1β at 4.668 ppm (Figure S2C). These are typical values for a Glc-unit in a lactose moiety, and as the Galβ1-4 H-1 signal is found at 4.461 (identical to that in Galβ6Galβ4Glc, see IV-3), the reducing end disaccharide can be identified as lactose. A characteristic H-6 signal of a 6-substituted Gal unit is seen at 4.05 ppm (2H by integration). This confirms the presence of two Galβ1-6 units. The fraction III-3 was therefore identified as Galβ6Galβ6Galβ4Glc.

The fraction III-4 1H-NMR spectrum was identical to that of fraction IV-4, and therefore this fraction was identified as Galβ3Galβ3Glc. Similarly, the fraction III-5 1H-NMR spectrum was identical to that of fraction IV-5, and this fraction was thus identified as Galβ3Galβ4Glc (spectra not shown).

The fraction II-1 was identified as a (HexNAc)1(Hex)3 tetrasaccharide by MALDI-TOF mass spectrometry (m/z 730.2, [M+Na]+) (not shown), and the 1H-NMR spectrum (Figure S3A) shows clear similarity to that of GalNAcα1-3Galβ1-4Glc (fraction III-1). Compared to the GalNAcα3Galβ4Glc spectrum, an additional β-glycosidic signal at 4.446 ppm is present in fraction II-1 spectrum. This is a typical value for a H-1 of Galβ1-6 unit. The downfield shift of GalNAc H-5 signal, from 4.195 to 4.396, strongly implies that the Galβ1-6 unit is linked to the GalNAc residue. The probable structure of fraction II-1 is thus Galβ6GalNAcα3Galβ4Glc. The nature of the fraction II-1 material was further studied using periodate oxidation, which oxidizes groups of vicinal hydroxyl units to aldehydes, whereby the C-C bond is cleaved. When coupled to MS analysis, this reaction is a very powerful tool to analyze the linkage patterns of oligosaccharides. The mass of the oxidized/reduced product is 623.3. Substitution of the penultimate GalNAc unit by a Galβ1-4 or Galβ1-3 residue would yield a product with mass 621.3. MALDI-TOF MS revealed a major oxidized/reduced product at m/z 646.2, [M+Na]+, (not shown), thus confirming the expected tetrasaccharide structure.

The 1H-NMR spectrum of fraction II-2 was identical to that of fraction III-3 (not shown), and the component was therefore identified as Galβ6Galβ6Galβ4Glc. The 1H-NMR spectrum (not shown) of fraction II-3 was identical to that of fraction IV-5. Thus, fraction II-3 was identified as Galβ3Galβ4Glc.

The fraction II-4 was identified as a (Hex)4 tetrasaccharide by MALDI-TOF mass spectrometry (m/z 689.2, [M+Na]+) (not shown). The 1H-NMR spectrum (Figure S3B) shows typical reducing end Glc-unit signals H-1α at 5.223 ppm and H-1β at 4.670 ppm, which are typical values for a Glc-unit in a lactose moiety. The Galβ1-4 H-1 signal is found at 4.481. In addition, two probable Gal H-1 signals were observed at 4.518 ppm and at 4.612 ppm. The spectrum reveals a characteristic H-6 signal of a 6-substituted Gal unit at 4.064 ppm (1H by integration). In addition, a typical H-4 signal (1H by integration) for a 3-substituted Gal-unit was observed at 4.236 ppm. These data suggest that II-4 is either Galβ3Galβ6Galβ4Glc or Galβ6Galβ3Galβ4Glc. Reference spectra for these substances are not available, but H-1 of terminal Galβ1-6 units typically resonates well below 4.5 ppm, while a substitution causes a clear downfield shift of the signal (cf. Galβ6Galβ4Glc and Galβ6Galβ6Galβ4Glc). The fraction II-4 structure was thus tentatively identified as Galβ3Galβ6Galβ4Glc. The II-4 component was subjected to periodate oxidation and MS/MS analysis for more detailed structural characterization, confirming that fraction II-4 is the tetrasaccharide Galβ3Galβ6Galβ4Glc.

The fraction II-5 was identified as a (Hex)4 tetrasaccharide by MALDI-TOF mass spectrometry (m/z 689.2, [M+Na]+) (not shown). The 1H-NMR spectrum (Figure S3C) shows typical lactose reducing end Glc-unit signals H-1α at 5.225 ppm, and H-1β at 4.667 ppm. Three probable Gal H-1 signals were observed at 4.432 ppm, 4.511 ppm and 4.622 ppm. A characteristic H-6 signal of a 6-substituted Gal unit is seen at 4.040 ppm (1H by integration). In addition, a typical H-4 signal (1H by integration) of a 3-substituted Gal-unit was observed at 4.223 ppm. The H-1 signal at 4.432 ppm implies that this tetrasaccharide contains a terminal Galβ1-6 residue. This would yield Galβ6Galβ3Galβ4Glc as the II-5 structure. To confirm the II-5 structure, the component was subjected to periodate oxidation and MS/MS analysis, confirming that the assignment of the NMR data was correct and the fraction II-5 can be identified as Galβ6Galβ3Galβ4Glc.

The fraction II-6 was identified as a (Hex)4 tetrasaccharide by MALDI-TOF mass spectrometry (m/z 689.2, [M+Na]+) (not shown). The 1H-NMR spectrum (Figure S3D) shows typical lactose reducing end Glc-unit signals H-1α at 5.224 ppm and H-1β at 4.666 ppm. The Galβ1-4 H-1 signal is found at 4.512 ppm, a value very similar to that in the trisaccharide Galβ3Galβ4Glc. A terminal Galβ1-3 unit is implied by the H-1 signal at 4.618 ppm. Another Galβ1-3 H-1 signal is observed at 4.678 ppm. H-4 signals of 3-substituted Gal-units arising from two protons are observed at 4.199 ppm. This analysis confirmed that fraction II-6 is the tetrasaccharide Galβ3Galβ3Galβ4Glc.

## References

[1] Backhed F., Ley R.E., Sonnenburg J.L., Peterson D.A., Gordon J.I. (2005). Host-bacterial mutualism in the human intestine. Science.

[2] Geuking M.B., Koller Y., Rupp S., McCoy K.D. (2014). The interplay between the gut microbiota and the immune system. Gut Microbes.

[3] Glendinning L., Free A. (2014). Supra-organismal interactions in the human intestine. Front. Cell. Infect. Microbiol..

[4] Illiano P., Brambilla R., Parolini C. (2020). The mutual interplay of gut microbiota, diet and human disease. FEBS J..

[5] Rooks M.G., Garrett W.S. (2016). Gut microbiota, metabolites and host immunity. Nat. Rev. Immunol..

[6] Soderholm A.T., Pedicord V.A. (2019). Intestinal epithelial cells: At the interface of the microbiota and mucosal immunity. Immunology.

[7] Chu H., Mazmanian S.K. (2013). Innate immune recognition of the microbiota promotes host-microbial symbiosis. Nat. Immunol..

[8] Sellge G., Kufer T.A. (2015). PRR-signaling pathways: Learning from microbial tactics. Semin. Immunol..

[9] Blacher E., Levy M., Tatirovsky E., Elinav E. (2017). Microbiome-modulated metabolites at the interface of host immunity. J. Immunol..

[10] Haase S., Haghikia A., Wilck N., Muller D.N., Linker R.A. (2018). Impacts of microbiome metabolites on immune regulation and autoimmunity. Immunology.

[11] Hills R.D., Pontefract B.A., Mishcon H.R., Black C.A., Sutton S.C., Theberge C.R. (2019). Gut microbiome: Profound implications for diet and disease. Nutrients.

[12] Stinson L.F. (2019). Establishment of the early-life microbiome: A dohad perspective. J. Dev. Orig. Health Dis..

[13] Floch M.H., Ringel Y., Walker W.A. (2016). The Microbiota in Gastrointestinal Pathophysiology: Implications for Human Health, Prebiotics, Probiotics, and Dysbiosis.

[14] Sanders M.E., Merenstein D.J., Reid G., Gibson G.R., Rastall R.A. (2019). Probiotics and prebiotics in intestinal health and disease: From biology to the clinic. Nat. Rev. Gastroenterol. Hepatol..

[15] Asahara T., Nomoto K., Shimizu K., Watanuki M., Tanaka R. (2001). Increased resistance of mice to salmonella enterica serovar typhimurium infection by synbiotic administration of bifidobacteria and transgalactosylated oligosaccharides. J. Appl. Microbiol..

[16] Childs C.E., Roytio H., Alhoniemi E., Fekete A.A., Forssten S.D., Hudjec N., Lim Y.N., Steger C.J., Yaqoob P., Tuohy K.M. (2014). Xylo-oligosaccharides alone or in synbiotic combination with bifidobacterium animalis subsp. Lactis induce bifidogenesis and modulate markers of immune function in healthy adults: A double-blind, placebo-controlled, randomised, factorial cross-over study. Br. J. Nutr..

[17] Le Leu R.K., Hu Y., Brown I.L., Woodman R.J., Young G.P. (2010). Synbiotic intervention of bifidobacterium lactis and resistant starch protects against colorectal cancer development in rats. Carcinogenesis.

[18] Roller M., Pietro Femia A., Caderni G., Rechkemmer G., Watzl B. (2004). Intestinal immunity of rats with colon cancer is modulated by oligofructose-enriched inulin combined with lactobacillus rhamnosus and bifidobacterium lactis. Br. J. Nutr..

[19] Umeki M., Oue K., Mochizuki S., Shirai Y., Sakai K. (2004). Effect of lactobacillus rhamnosus ky-3 and cellobiose as synbiotics on lipid metabolism in rats. J. Nutr. Sci. Vitam..

[20] Dilli D., Aydin B., Fettah N.D., Ozyazici E., Beken S., Zenciroglu A., Okumus N., Ozyurt B.M., Ipek M.S., Akdag A. (2015). The propre-save study: Effects of probiotics and prebiotics alone or combined on necrotizing enterocolitis in very low birth weight infants. J. Pediatr..

[21] Roller M., Rechkemmer G., Watzl B. (2004). Prebiotic inulin enriched with oligofructose in combination with the probiotics lactobacillus rhamnosus and bifidobacterium lactis modulates intestinal immune functions in rats. J. Nutr..

[22] Pothoulakis C., Lamont J.T. (2001). Microbes and microbial toxins: Paradigms for microbial-mucosal interactions II. The integrated response of the intestine to clostridium difficile toxins. Am. J. Physiol. Gastrointest. Liver Physiol..

[23] Padua D., Pothoulakis C. (2016). Novel approaches to treating clostridium difficile-associated colitis. Expert Rev. Gastroenterol. Hepatol..

[24] Vincent Y., Manji A., Gregory-Miller K., Lee C. (2015). A review of management of clostridium difficile infection: Primary and recurrence. Antibiotics.

[25] Goldenberg J.Z., Yap C., Lytvyn L., Lo C.K., Beardsley J., Mertz D., Johnston B.C. (2017). Probiotics for the prevention of clostridium difficile-associated diarrhea in adults and children. Cochrane Database Syst. Rev..

[26] Madoff S.E., Urquiaga M., Alonso C.D., Kelly C.P. (2020). Prevention of recurrent clostridioides difficile infection: A systematic review of randomized controlled trials. Anaerobe.

[27] McFarland L.V. (2015). Probiotics for the primary and secondary prevention of *C. Difficile* infections: A meta-analysis and systematic review. Antibiotics.

[28] Szajewska H., Kolodziej M. (2015). Systematic review with meta-analysis: Saccharomyces boulardii in the prevention of antibiotic-associated diarrhoea. Aliment. Pharmacol. Ther..

[29] Vernaya M., McAdam J., Hampton M.D. (2017). Effectiveness of probiotics in reducing the incidence of clostridium difficile-associated diarrhea in elderly patients: A systematic review. JBI Database Syst. Rev. Implement. Rep..

[30] Euler A.R., Mitchell D.K., Kline R., Pickering L.K. (2005). Prebiotic effect of fructo-oligosaccharide supplemented term infant formula at two concentrations compared with unsupplemented formula and human milk. J. Pediatr. Gastroenterol. Nutr..

[31] Lewis S., Burmeister S., Cohen S., Brazier J., Awasthi A. (2005). Failure of dietary oligofructose to prevent antibiotic-associated diarrhoea. Aliment. Pharmacol. Ther..

[32] Smilowitz J.T., Lemay D.G., Kalanetra K.M., Chin E.L., Zivkovic A.M., Breck M.A., German J.B., Mills D.A., Slupsky C., Barile D. (2017). Tolerability and safety of the intake of bovine milk oligosaccharides extracted from cheese whey in healthy human adults. J. Nutr. Sci..

[33] Urashima T., Saito T., Nakamura T., Messer M. (2001). Oligosaccharides of milk and colostrum in non-human mammals. Glycoconj. J..

[34] Zivkovic A.M., Barile D. (2011). Bovine milk as a source of functional oligosaccharides for improving human health. Adv. Nutr..

[35] Rastall R.A. (2010). Functional oligosaccharides: Application and manufacture. Annu. Rev. Food Sci. Technol..

[36] Sako T., Matsumoto K., Tanaka R. (1999). Recent progress on research and applications of non-digestible galacto-oligosaccharides. Int. Dairy J..

[37] Coulier L., Timmermans J., Bas R., Van Den Dool R., Haaksman I., Klarenbeek B., Slaghek T., Van Dongen W. (2009). In-depth characterization of prebiotic galacto-oligosaccharides by a Combination of Analytical Techniques. J. Agric. Food Chem..

[38] Yanahira S., Kobayashi T., Suguri T., Nakakoshi M., Miura S., Ishikawa H., Nakajima I. (1995). Formation of oligosaccharides from lactose by bacillus circulans beta-galactosidase. Biosci. Biotechnol. Biochem..

[39] Van Leeuwen S.S., Kuipers B.J.H., Dijkhuizen L., Kamerling J.P. (2014). (1)H NMR Analysis of the lactose/β-galactosidase-derived Galacto-Oligosaccharide Components of Vivinal® GOS Up to DP5. Carbohydr. Res..

[40] Austin S., Benet T., Michaud J., Cuany D., Rohfritsch P. (2014). Determination of beta -galactooligosaccharides by liquid chromatography. Int. J. Anal. Chem..

[41] Dumortier V., Montreuil J., Bouquelet S. (1990). Primary structure of ten galactosides formed by transglycosylation during lactose hydrolysis by bifidobacterium bifidum. Carbohydr. Res..

[42] Chen S., Wei D., Hu Z. (2001). Synthesis of galacto-oligosaccharides by immobilized Bacillus stearothermophilus. Wei Sheng Wu Xue Bao.

[43] Drakoularakou A., Tzortzis G., Rastall R.A., Gibson G.R. (2010). A double-blind, placebo-controlled, randomized human study assessing the capacity of a novel galacto-oligosaccharide mixture in reducing travellers’ diarrhoea. Eur. J. Clin. Nutr..

[44] Krumbeck J.A., Maldonado-Gomez M.X., Martinez I., Frese S.A., Burkey T.E., Rasineni K., Ramer-Tait A.E., Harris E.N., Hutkins R.W., Walter J. (2015). In Vivo selection to identify bacterial strains with enhanced ecological performance in synbiotic applications. Appl. Environ. Microbiol..

[45] Krumbeck J.A., Rasmussen H.E., Hutkins R.W., Clarke J., Shawron K., Keshavarzian A., Walter J. (2018). Probiotic bifidobacterium strains and galactooligosaccharides improve intestinal barrier function in obese adults but show no synergism when used together as synbiotics. Microbiome.

[46] Simeoni U., Berger B., Junick J., Blaut M., Pecquet S., Rezzonico E., Grathwohl D., Sprenger N., Brussow H., Szajewska H. (2016). Gut microbiota analysis reveals a marked shift to bifidobacteria by a starter infant formula containing a synbiotic of bovine milk-derived oligosaccharides and bifidobacterium animalis subsp. Lactis CNCM I-3446. Environ. Microbiol..

[47] So D., Whelan K., Rossi M., Morrison M., Holtmann G., Kelly J.T., Shanahan E.R., Staudacher H.M., Campbell K.L. (2018). Dietary fiber intervention on gut microbiota composition in healthy adults: A systematic review and meta-analysis. Am. J. Clin. Nutr..

[48] Swanson K.S., De Vos W.M., Martens E.C., Gilbert J.A., Menon R.S., Soto-Vaca A., Hautvast J., Meyer P.D., Borewicz K., Vaughan E.E. (2020). Effect of fructans, prebiotics and fibres on the human gut microbiome assessed by 16s rrna-based approaches: A review. Benef. Microbes.

[49] Barboza M., Sela D.A., Pirim C., Locascio R.G., Freeman S.L., German J.B., Mills D.A., Lebrilla C.B. (2009). Glycoprofiling bifidobacterial consumption of galacto-oligosaccharides by mass spectrometry reveals strain-specific, preferential consumption of glycans. Appl. Environ. Microbiol..

[50] Tzortzis G., Goulas A.K., Gibson G.R. (2005). Synthesis of prebiotic galactooligosaccharides using whole cells of a novel strain, bifidobacterium bifidum ncimb 41171. Appl. Microbiol. Biotechnol..

[51] Tzortzis G., Goulas A.K., Gee J.M., Gibson G.R. (2005). A novel galactooligosaccharide mixture increases the bifidobacterial population numbers in a continuous in vitro fermentation system and in the proximal colonic contents of pigs in vivo. J. Nutr..

[52] Di Bella S., Ascenzi P., Siarakas S., Petrosillo N., Di Masi A. (2016). Clostridium difficile toxins a and b: Insights into pathogenic properties and extraintestinal effects. Toxins.

[53] Tao Y., Drabik K.A., Waypa T.S., Musch M.W., Alverdy J.C., Schneewind O., Chang E.B., Petrof E.O. (2006). Soluble factors from lactobacillus gg activate mapks and induce cytoprotective heat shock proteins in intestinal epithelial cells. Am. J. Physiol. Cell Physiol..

[54] Liu T.S., Musch M.W., Sugi K., Walsh-Reitz M.M., Ropeleski M.J., Hendrickson B.A., Pothoulakis C., Lamont J.T., Chang E.B. (2003). Protective role of hsp72 against clostridium difficile toxin a-induced intestinal epithelial cell dysfunction. Am. J. Physiol. Cell Physiol..

[55] Musch M.W., Kaplan B., Chang E.B. (2001). Role of increased basal expression of heat shock protein 72 in colonic epithelial c2BBE adenocarcinoma cells. Cell Growth Differ..

[56] Yan F., Polk D.B. (2002). Probiotic bacterium prevents cytokine-induced apoptosis in intestinal epithelial cells. J. Biol. Chem..

[57] Yan F., Polk D.B. (2012). Characterization of a probiotic-derived soluble protein which reveals a mechanism of preventive and treatment effects of probiotics on intestinal inflammatory diseases. Gut Microbes.

[58] Yan F., Polk D.B. (2012). Lactobacillus Rhamnosus GG: An updated strategy to use microbial products to promote health. Funct. Food Rev. (Print).

[59] Chen X., Kokkotou E.G., Mustafa N., Bhaskar K.R., Sougioultzis S., O’Brien M., Pothoulakis C., Kelly C.P. (2006). Saccharomyces boulardii inhibits ERK1/2 mitogen-activated protein kinase activation both in vitro and in vivo and protects against clostridium difficile toxin A-induced enteritis. J. Biol. Chem..

[60] Duncan P.I., Fotopoulos G., Pasche E., Porta N., Masserey Elmelegy I., Sanchez-Garcia J.L., Bergonzelli G.E., Corthesy-Theulaz I. (2009). Yeast, beef and pork extracts counteract clostridium difficile toxin a enterotoxicity. FEMS Microbiol. Lett..

[61] Na X., Kim H., Moyer M.P., Pothoulakis C., LaMont J.T. (2008). Gp96 is a human colonocyte plasma membrane binding protein for clostridium difficile toxin a. Infect. Immun..

[62] Akashi-Takamura S., Miyake K. (2008). TLR accessory molecules. Curr. Opin. Immunol..

[63] Yang F., Wang J., Li X., Ying T., Qiao S., Li D., Wu G. (2007). 2-DE and MS analysis of interactions between Lactobacillus fermentum I5007 and intestinal epithelial cells. Electrophoresis.

[64] Segers M.E., Lebeer S. (2014). Towards a better understanding of lactobacillus rhamnosus GG—Host interactions. Microb. Cell Factories.

