# Supplementary material for: Structure and Function of Bovine Whey Derived Oligosaccharides Showing Synbiotic Epithelial Barrier Protective Properties
Source: Nutrients. 2020 Jul 6;12(7):2007. doi: 10.3390/nu12072007 (PMC7400958; doi:10.3390/nu12072007)
Supplement: Supplementary file 1 [file nutrients-12-02007-s001.pdf]

Characterization of the isolated oligosaccharides.

Table S1. Proton chemical shift values (ppm) of GOS disaccharide and trisaccharide components.

	**IV-2**	**IV-1**	**IV-3/III-1**	**IV-4/III-4**	**IV-5/III-5/II-3**	**III-1b**
	**Galβ4Glc**	**Galβ6Gal**	**Galβ6Galβ4Glc**	**Galβ3Galβ3Glc**	**Galβ3Galβ4Glc**	**GalNAcα3Galβ4Glc**
Glc						
H1α	5.223		5.222	5.222	5.224	5.225
H1β	4.665		4.669	4.663	4.666	4.667
Gal						
H1α		5.265				
H1β		4.594				
Gal(4)						
H1	4.452		4.461		4.511	4.514
H4					4.197	4.111
H6			4.07			
Gal(6)						
H1α		4.444				
H1β		4.454				
Gal(3)						
H1				4.484		
H4				4.194		
Gal(6,4)						
H1			4.485			
Gal(3,4)						
H1					4.614	
Gal(3,3)						
H1				4.602		
GalNAc(3,4)						
H1						5.082
H2						4.231
H5						4.195

Table S2. Proton chemical shift values (ppm) of GOS tetrasaccharide components.

	**III-3/II-2**	**II-4**	**II-5**	**II-6**
	**Galβ6Galβ6Galβ4 Glc**	**Galβ3Galβ6Galβ4 Glc**	**Galβ6Galβ3Galβ4 Glc**	**Galβ3Galβ3Galβ4 Glc**
Glc				
H1α	5.222	5.223	5.225	5.224
H1β	4.668	4.670	4.667	4.666
Gal(4)				
H1	4.461	4.481	4.511	4.512
H4			4.223	4.199
H6	4.05	4.064		
Gal(6,4)				
H1	4.501	4.518		
H4		4.236		
H6	4.05			
Gal(6,6,4)				
H1	4.453			
Gal(3,4)				
H1			4.622	4.678
H4				4.199
H6			4.040	
Gal(6,3,4)				
H1			4.432	
Gal(3,6,4)				
H1		4.612		
Gal(3,3,4)				
H1				4.618

Table S3. Proton chemical shift values (ppm) of GOS tetrasaccharide components.

	II-1	III-2
	**Galβ6GalNAcα3Galβ4 Glc**	**Galβ6Galβ6Galβ6Glc**
Glc		
H1α	5.224	5.223
H1β	4.666	4.684
H6α		4.213
H6β		4.287
Gal(4)		
H1	4.511	
H4	4.076	
Gal(6)		
H1α		4.444
H1β		4.455
H6		4.07
GalNAc(3,4)		
H1	5.068	
H2	4.225	
H5	4.396	
Gal(6,6)		
H1		4.514
H6		4.07
Gal(6,3,4)		
H1	4.446	
Gal(6,6,6)		
H1		4.506

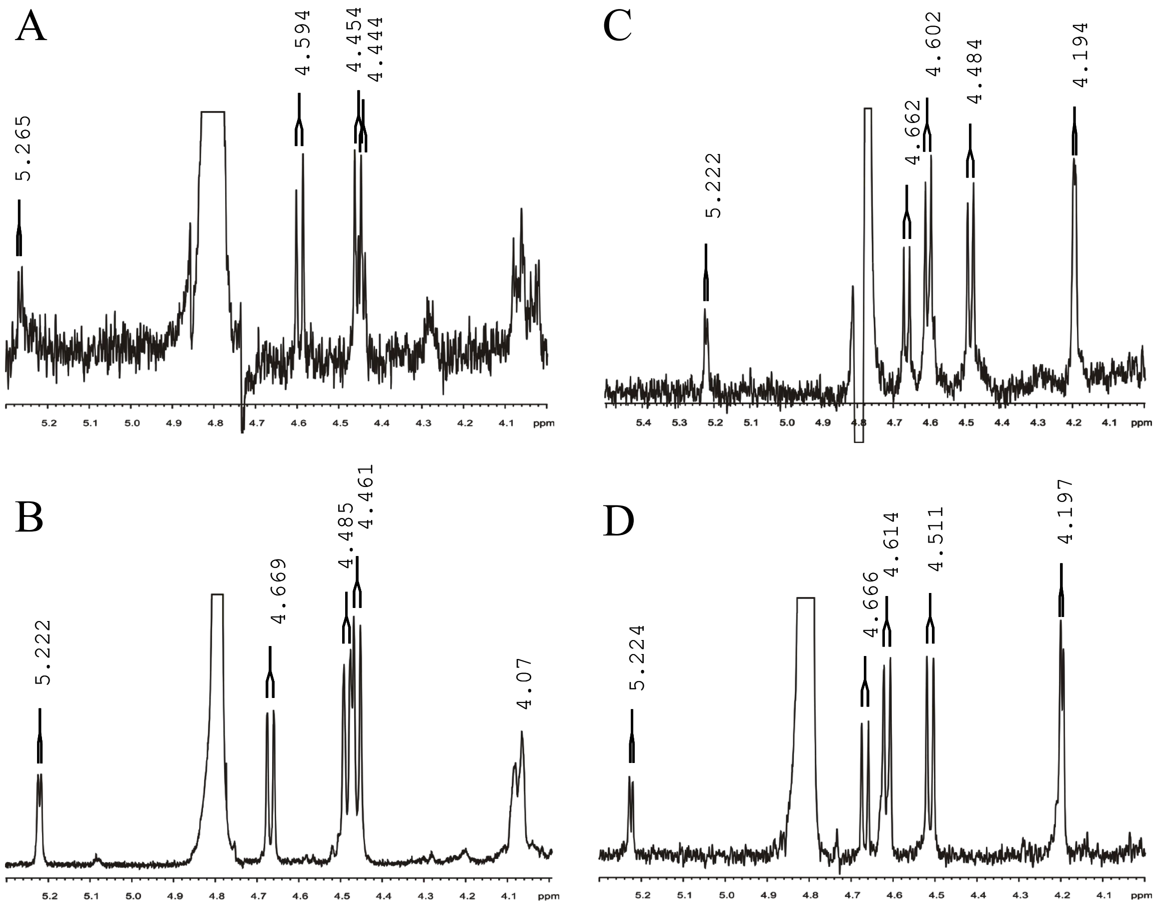

Figure S1. 1H-NMR spectra of GOS components from fraction IV. (**A**) IV-1, (**B**) IV-3, (**C**) IV-4 and (**D**) IV-5. See text and Table 1 for assignments.

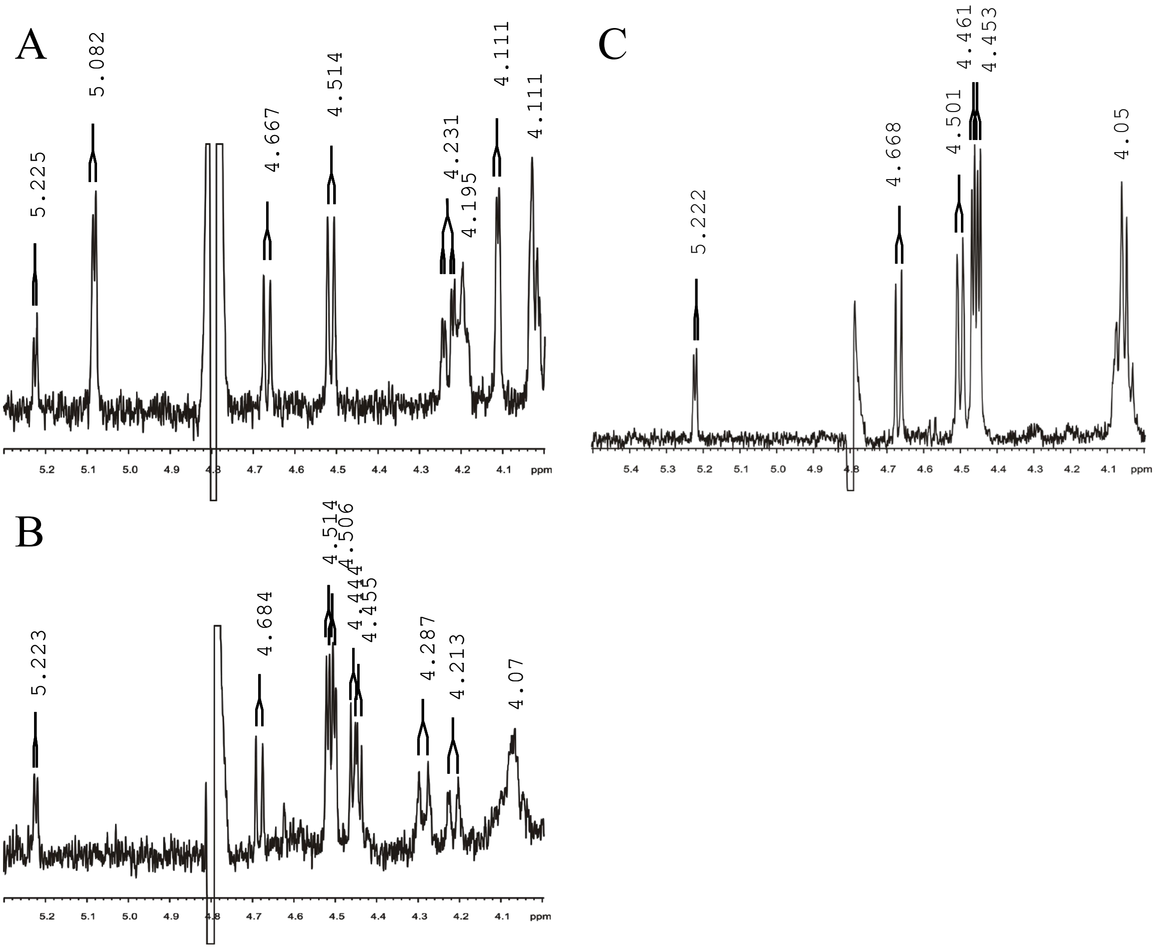

Figure S2. 1H-NMR spectra of GOS components from fraction III. (**A**) III-1b, (**B**) III-2 and (**C**) III-3. See text and Tables S1–S3 for assignments.

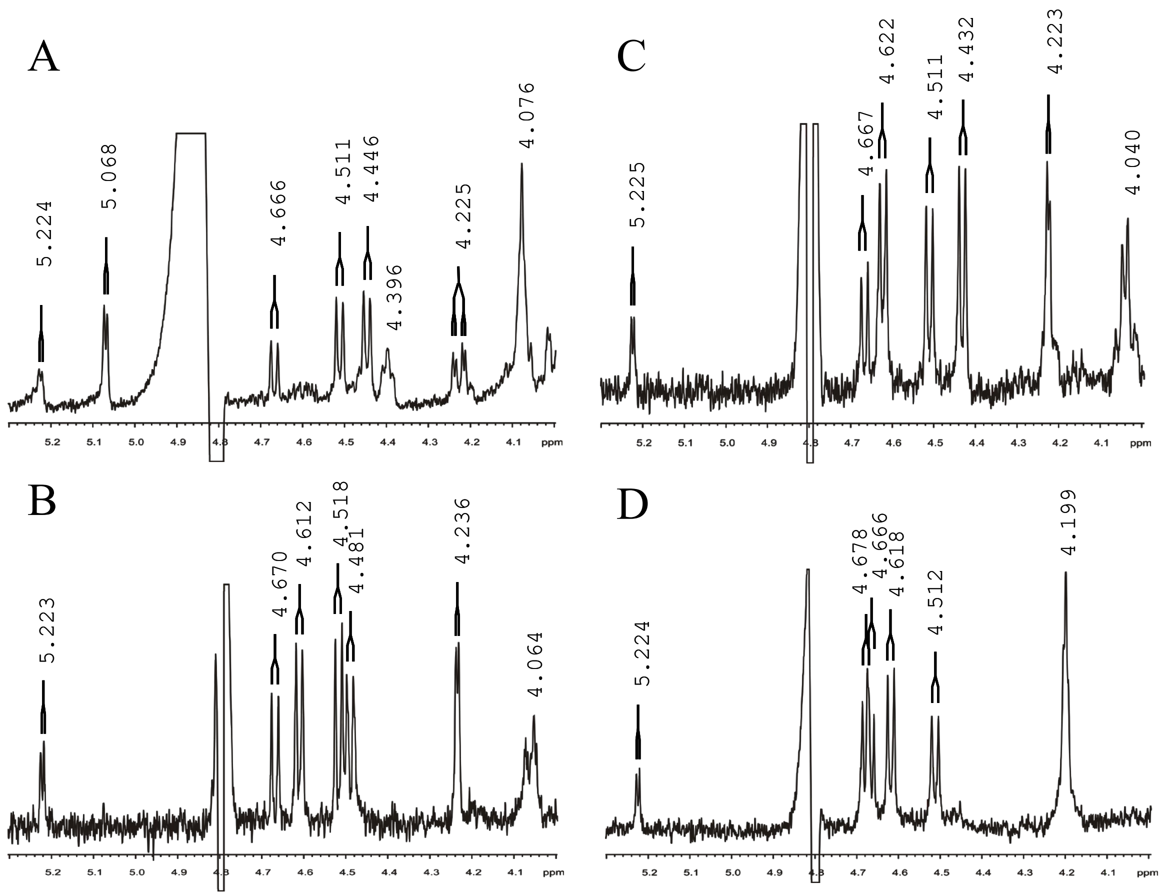

Figure S3. 1H-NMR spectra of GOS components from fraction II. (**A**) II-1, (**B**) II-4, (**C**) II-5 and (**D**) II-6. See text and Tables 2 and 3 for assignments.
